# A short guide on blue fluorescent proteins: limits and perspectives

**DOI:** 10.1007/s00253-024-13012-w

**Published:** 2024-02-14

**Authors:** Pil-Won Seo, Geun-Joong Kim, Jeong-Sun Kim

**Affiliations:** 1 Department of Chemistry, Chonnam National University, Gwangju, 61186 Republic of Korea; 2 Department of Biological Sciences, Chonnam National University, Gwangju, 61186 Republic of Korea

**Keywords:** Fluorescent protein, Blue fluorescent protein, GFP, BFP

## Abstract

**Abstract:**

The advent of the so-called colorful biology era is in line with the discovery of fluorescent proteins (FPs), which can be widely used to detect the intracellular locations of macromolecules or to determine the abundance of metabolites in organelles. The application of multiple FPs that emit different spectra and colors could be implemented to precisely evaluate cellular events. FPs were initially established with the emergence of the green fluorescent protein (GFP) from jellyfish. Red fluorescent proteins (RFPs) from marine anemones and several corals adopt fluorescent chromophores that are similar to GFP. Chromophores of GFP and GFP-like FPs are formed through the oxidative rearrangement of three chromophore-forming residues, thereby limiting their application to only oxidative environments. Alternatively, some proteins can be fluorescent upon their interaction with cellular prosthetic cofactors and, thus, work in aerobic and anaerobic conditions. The modification of an NADPH-dependent blue fluorescent protein (BFP) also expanded its application to the quantization of NADPH in the cellular environment. However, cofactor-dependent BFPs have an intrinsic weakness of poor photostability with a high fluorescent background. This review explores GFP-derived and NADPH-dependent BFPs with a focus on NADPH-dependent BFPs, which might be technically feasible in the near future upon coupling with two-photon fluorescence microscopy or nucleic acid-mimickers.

**Key points:**

*• Oxidation-dependent GFP-like BFPs and redox-free NADPH-dependent BFPs*

*• GFPs of weak photostability and intensity with a high fluorescent background*

*• Real-time imaging using mBFP under two-photon fluorescence microscopy*

## Introduction

**Fig. 1:**
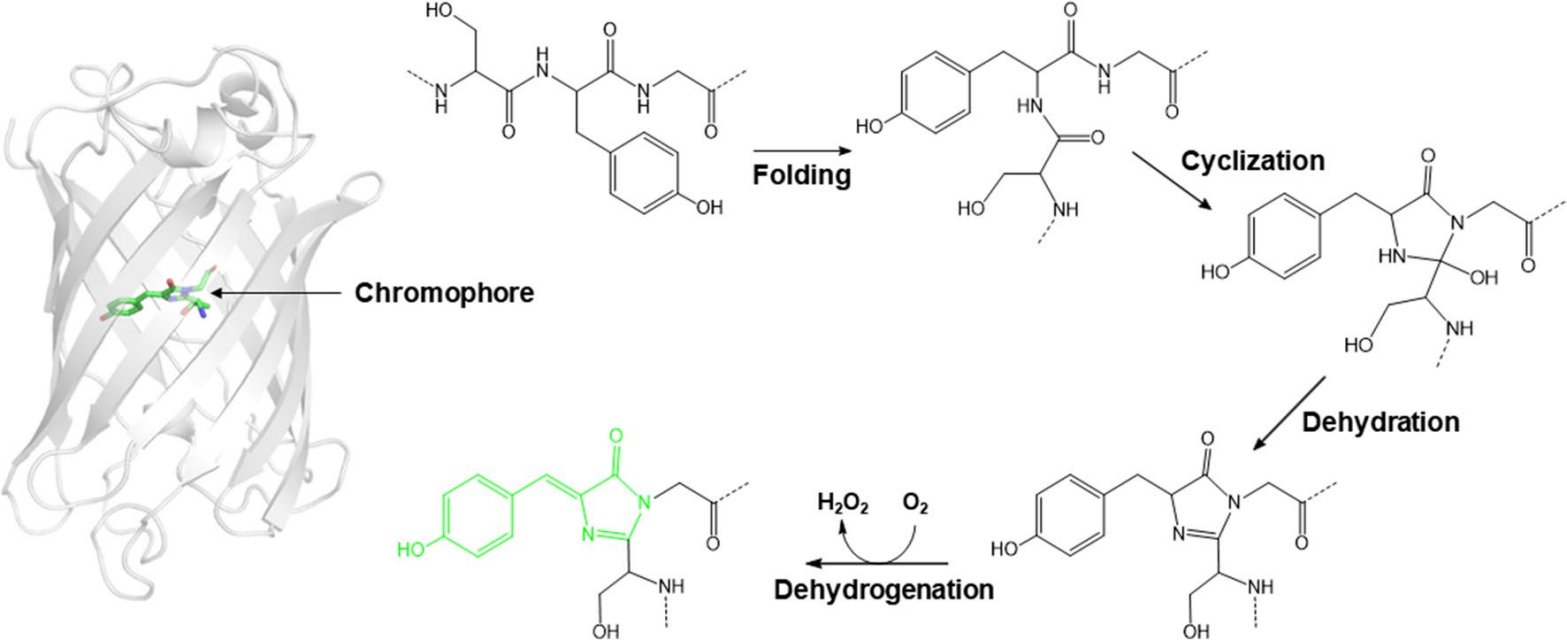
A widely accepted mechanism of chromophore formation for avGFP

**Fig. 2:**
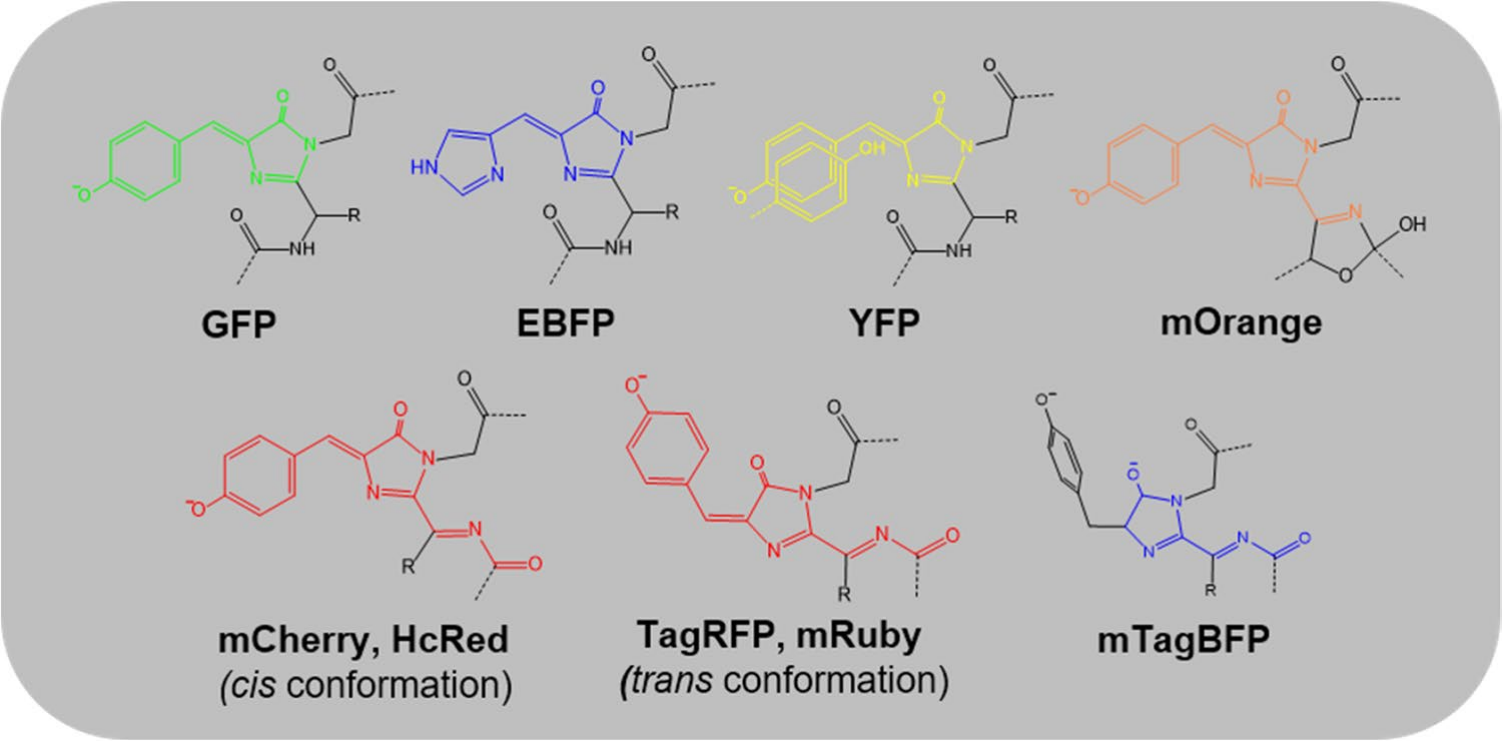
Chemical structures of selected GFP-like proteins. The π-conjugation system responsible for fluorescence emission is colored to correspond with the color of fluorescence emission

Fluorescent proteins (FPs) are a group of proteins that can absorb light at a specific wavelength and then emit light at a longer wavelength, a phenomenon known as fluorescence. Among FPs, the initially reported blue fluorescent protein (BFP) is closely related to green fluorescent protein (GFP). In the early 1960s, two scientists at the University of Washington tried to isolate a calcium ion–dependent bioluminescent protein (aequorin) from the *Aequorea victoria* jellyfish. During this trial, they discovered one additional co-migrating protein with aequorin that produced green fluorescence upon illumination with ultraviolet light. Hence, this additional protein was named GFP. Subsequent structural studies have revealed that GFP from *A. victoria* (avGFP) comprises an 11-stranded β-barrel with an axial helix in the central hole of the β-barrel (Ormo et al. 1996). Three consecutive amino acids (Ser65, Tyr66, and Gly67) located on the central axial helix rearrange and form a chromophore, 4-(p-hydroxybenzylidene)-5-imidazolinone (p-HBI), through the processes of cyclization, dehydration, and oxidation (Fig. 1) (Kong et al. 2020). The wild-type (wt) avGFP exhibits an absorption spectrum with maximal excitation at 397 nm and a minor secondary peak at 476 nm. Further dynamics studies revealed that its fluorescence was related to the deprotonation of the Tyr66 residue (Chattoraj et al. 1996). However, the significantly higher extinction coefficient at near-UV wavelengths and the low quantum yield hindered its application in cellular imaging. Thus, mutagenesis has been implemented to yield GFP derivatives that emit fluorescence corresponding from the blue to yellow regions in the visible spectrum (Tsien 1998). The GFP-driven BFP was developed by mutation of the Tyr66 residue (Fig. 2). This mutation effectively changed the structure of the chromophore, altering its optical properties and shifting its fluorescence emission from green to blue. The development of BFP was a significant milestone as it expanded the utility of fluorescent proteins beyond GFP. FPs emitting within the orange and red spectral regions have also been developed from marine anemones, *Discosoma striata*, and several reef corals from the Anthozoa class (Fig. 2) (Verkhusha and Lukyanov 2004). Moreover, all the aforementioned GFPs and related proteins do not require any additional exogenous factors to emit fluorescence.

A combination of FPs that emit different spectral regions can provide a better background for deducing intracellular dynamics and protein–protein interactions because a crossing point of FPs with different spectra can minimize the probable misinterpretations that might be derived from a single FP. Furthermore, GFP-based FPs work in a strictly aerobic environment. BFP, which is a reported GFP derivative (Y66H, Y145F), with an emission maximum of 445 nm, also has the same status in the application field. Therefore, FPs that function in anaerobic conditions can expand the detection of cellular events. Recently, numerous proteins have been characterized that increase the intensity of intrinsic fluorescent cofactors, such as nicotine adenine dinucleotide (NAD(P)H) and flavin adenine dinucleotide (FAD) (Hsu et al. 1967; Hwang et al. 2012; Lakowicz et al. 1992; Li and Lin 1996; Su et al. 2001). Some NAD(P)H-binding proteins have been used to enhance the intensity of the blue spectrum by NAD(P)H and, thus, have been named NAD(P)H cofactor-dependent blue fluorescent proteins (BFPs). Unlike those from GFP derivatives, its origin of fluorescence is dependent on a factor being supplied exogenously. The increasing fluorescence of the bound NAD(P)H was supposedly related to its conformational change following the interaction with protein atoms (Velick 1958).

This review (1) briefly summarizes GFP-derived BFPs reported so far, (2) intensively describes the development of NADPH-dependent BFPs, and (3) explores in depth the limitations and perspectives of BFPs in the intracellular environment. This is discerned by some reviews that mostly described the properties of GFP-derived BFPs and their usages (Chen et al. 2023; Nienhaus and Nienhaus 2022; Wang et al. 2023).

## Blue fluorescent proteins (BFPs) of β-barrel variants

### GFP-derived BFPs

Replacing the Tyr66 residue with histidine (Y66H) in the three chromophore-forming residues in avGFP changed its spectra from green to blue, thereby generating a GFP variant that emits blue fluorescence with an excitation and emission spectra at wavelengths of 384 nm and 448 nm, respectively. Therefore, this GFP derivative has been named blue fluorescent protein (BFP) (Table 1). However, the relatively small side chain in the histidine residue at this site conferred rather a high flexibility in the BFP chromophore to the remaining parts of the protein, which rendered the chromophore highly susceptible to photobleaching and resulted in a very low quantum yield. Concurrently, the short wavelength used to excite the BFP chromophore was not favorable since UV light at ~ 384 nm also influences the intrinsic fluorescent biomolecules, such as flavins, therefore limiting its use in cellular imaging experiments. As with other GFPs, the required long maturation time for forming the fluorescence chromophore is another hurdle for in vivo imaging.

**Table 1:** Properties of selected avGFP derivatives

Protein (acronym)	Ex/nm	Em/nm	EC (10^−3^ M^−1^ cm^−1^)	QY	Quaternary structure	Effective p*K*_a_	Brightness (× 10^−3^ M^−1^ cm^−1^)	Ref.
Green fluorescent proteins								
GFPuv	394	509	27.8	0.8	Monomer	–	22.2	Shimizu et al. (2017)
Blue fluorescent proteins								
BFP	384	448	21.0	0.24	Monomer*	–	5.0	(Tsien 1998)
EBFP	383	445	29.0	0.31	Monomer*	6.3	9.0	Patterson et al. (1997)
Azurite	384	448	26.2	0.55	Monomer*	5	14.4	Mena et al. (2006)
EBFP2	383 (386)	448 (448)	32.0 (39.0)	0.56 (0.53)	Monomer*	5.3(4.4)	17.9 (20.7)	Ai et al. (2007)
Sirius	355	424	15.0	0.24	Monomer*	< 3.0	3.6	Tomosugi et al. (2009)
mTagBFP	399	456	52.0	0.63	Monomer	2.7	32.8	Subach et al. (2008)
mTagBFP2	399 (400)	454 (454)	50.6 (76.0)	0.64 (0.48)	Monomer	2.7 (2.4)	32.4 (36.5)	Subach et al. (2011)

The peak excitation (Ex) and emission (Em) wavelengths, molar extinction coefficient (EC), quantum yield (QY), brightness, and physiological quaternary structure are listed. Values without parentheses are from original articles. Values indicated in parentheses are referenced from a paper (Cranfill et al. 2016)

*Signifies a weak dimer

To enhance the reduced brightness of blue fluorescence from the GFP-derived His-containing (Y66H) BFP chromophore, residues near the chromophore were modified to produce potentially enhanced BFPs (EBFPs). The addition of further mutations yielded EBFP variants, one of which included mutations at three individual sites (EBFP-T65S/V150I/V224R) and improved the brightness by two-fold, compared with EBFP. Interestingly, this derivative was also 40 times more photostable than its parent protein and was subsequently named Azurite (Mena et al. 2006). Azurite with irrespective excitation and emission spectra at 384 nm and 448 nm, respectively, was further modified to improve its solubility and folding kinetics at 37 °C (Sample et al. 2009), thereby resulting in another EBFP variant (EBFP2) with an excitation wavelength of 386 nm. The fluorescence intensity of EBFP2 is four times higher while presenting a photostability increase of 550 times, compared with EBFP. Thus, using EBFP2 in cellular imaging studies could provide better results than other GFP-driven derivatives. Detailed mutation points for engineering EBFP can be found in other reviews (Ai et al. 2007; Shaner et al. 2007).

Another BFP was yielded by substituting the Tyr66 residue in the chromophore of avGFP with phenylalanine. This BFP has shown spectral profiles (excitation and emission at 360 nm and 442 nm, respectively), which are rather different from those of EBFP, Azurite, and EBFP2 (Table 1). However, the fluorescence brightness of Y66F GFP was very low. Further studies using this Y66F derivative produced another variant (Sirius) that presents with a much brighter fluorescence than the parent Y66F derivative, possesses more photostability than EBFP2, and exhibits a reduced insensitivity to pH fluctuations (pH values ranging from 3 to 9). Nonetheless, the brightness of Sirius is still low—equivalent to only 10% of the EGFP brightness. However, its shortest emission wavelength (424 nm) can be compatible with fluorescence resonance energy transfer (FRET), although its excitation in the ultraviolet region (355 nm) can also prove problematic for long-term time-lapse imaging of living cells owing to problems associated with phototoxicity and autofluorescence.

### Red fluorescent proteins (RFPs) can be BFPs

Red fluorescent proteins (RFPs) emit a UV-VIS light that is depicted as red–orange following excitation by UV. The RFP structures are similar to the β-barrel structure of AvGFP. RFP chromophores are also formed by a tripeptide motif (Met–Tyr–Gly) in the internal axial helix of the β-barrel. The resulting chromophores are also p-HBI, which is excited by a laser at a wavelength of 488 nm or 532 nm and was readily detected at 588 nm. Indeed, small differences are present in the wavelengths of the various RFPs, such as TagRFP (555 vs. 584 nm) (Merzlyak et al. 2007), mCherry (587 vs. 610 nm) (Shaner et al. 2004), HcRed (592 vs. 645 nm) (Fradkov et al. 2002), and mRuby (558 vs. 605 nm) (Kredel et al. 2009). In contrast to the common monomeric structure of GFP (Tsien 1998), oligomeric states of RFPs are diverse, for example, monomeric TagRFP, mRuby, and mCherry or tetrameric HcRed. Derivatizations of orange and red fluorescent proteins from Anthozoa FPs were successful in generating other BFPs that were distinct from the GFP-driven BFPs of EBFPs, Azurite, and Sirius.

TagRFP, a GFP-like RFP, has a chromophore that is automatically rearranged by three residues: Met65, Tyr66, and Gly67. TagRFP has a spectral property of excitation at 555 nm and emission at 584 nm. Similar to Azurite, replacing the residues that interact with the chromophore-forming tripeptide changed its fluorescence from red to blue. This blue fluorescent TagRFP derivative exists as a monomeric protein in solution; thus, it is named mTagBFP (Subach et al. 2008). The fluorescent spectrum change for mTagBFP is related to a newly formed N-acylimine chromophore, which represents a modified version of p-HBI. The excitation/emission peaks for mTagBFP were observed at 399 nm and 456 nm, respectively. mTagBFP has exhibited superior brightness, faster chromophore maturation, and higher pH stability (Table 1) than the Aequorea FPs that have a histidine residue in the chromophore, for example, EBFP2. mTagBFP has shown less photostability than EBFP2 under exposure with an arc lamp yet higher stability with laser light. Therefore, owing to this property, FRET donors can use mTagBFP when it is coupled with green and yellow FPs from both Aequorea and Anthozoa, respectively (Day and Davidson 2009). Similarly, derivatizations of mCherry and HcRed1 also yielded BFPs when some of the chromophore-contacting residues were modified into others with Ex/Em wavelengths of 403/458 nm and 408/455 nm for the mCherry variant and the HcRed1 variant, respectively (Subach et al. 2008).

Structure-guided modifications to mTagBFP1 were performed to affect the spatial position of the Tyr residue in the chromophore-forming residues. The introduction of a single point mutation (I174A, mTagBFP2) in the protein increased its photostability by 1.5 times under illumination with an arc lamp, compared with mTagBFP1 (Subach et al. 2011), and the brightness of mTagBFP2 was also much higher in living cells than by mTagBFP. In spite of mutations at several points, mTagBFP2 still maintains other advantages over mTagBFP1, while its FRET efficiency is regarded as being better than mTagBFP. Therefore, it has been evaluated as the best current GFP-like BFP. Recently, two variants of mRuby3, named Electra1 and Electra2, were reported, and quantification of intracellular brightness showed that Electra1 and Electra2 were 2.3 and 2.1 times brighter than mTagBFP2 (Papadaki et al. 2022).

## NAD(P)H-dependent BFPs

**Fig. 3:**
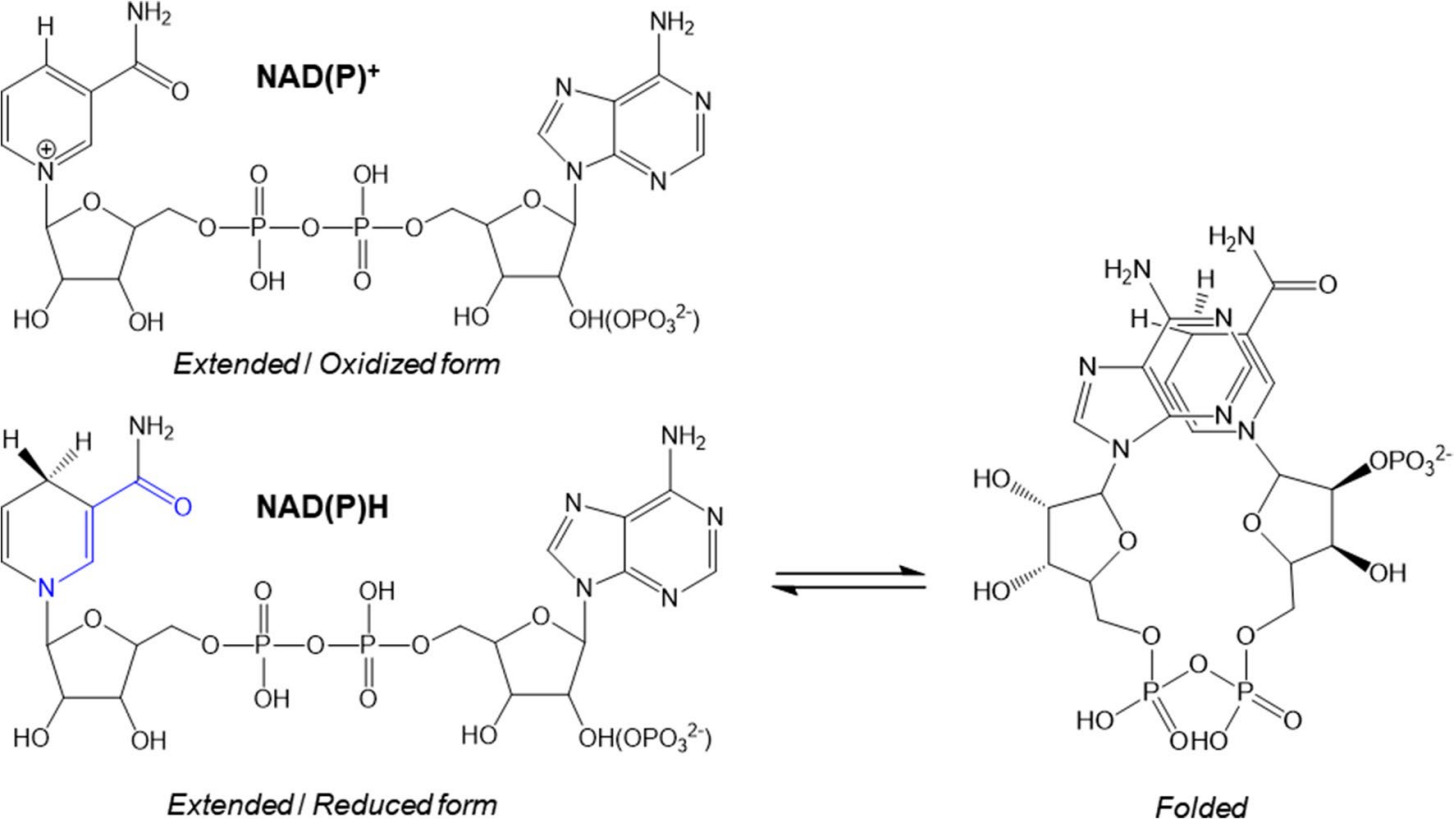
The two conformations of NAD(P)H. NAD(P)^+^ differs from NAD(P)H by the added hydride ion to the nicotinamide ring. Absorption of light by NAD(P)H causes a shift in electron density from the nicotinamide nitrogen toward the oxygen of the amide group (shown in blue)

**Fig. 4:**
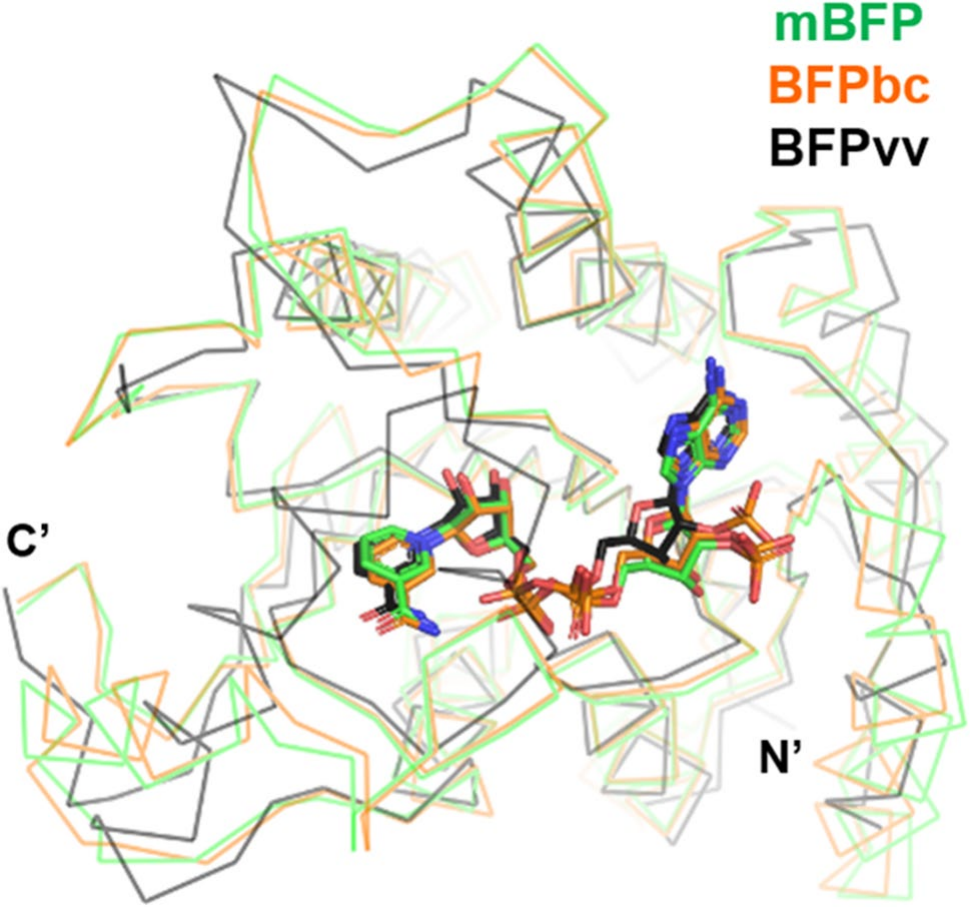
Structural comparison of three NAD(P)H-dependent blue fluorescent proteins. Monomeric NADPH-bound BFP from metagenomic library (mBFP; PDB ID 6J7U), NADP-bound BFP from *Burkholderia cenocepacia* J2315 (BFPbc; PDB ID 5U2W), and NADPH-bound BFP from *Vibrio vulnificus* (BFPvv; PDB ID 3P19) are depicted by alternating colors. Bound NADPH (mBFP and BFPvv) and NADP (BFPbc) are displayed as stick models

NAD(P)H is an intrinsic fluorescent molecule with two strong UV-VIS absorption bands in aqueous solution at 260 and 340 nm. The absorption band at 260 nm is related to the transition within the adenylyl moiety, while the band at 340 nm is due to the dihydronicotinamide moiety. The origin of the amplified NAD(P)H fluorescence (Fig. 3) is also related to dihydronicotinamide. Upon returning the activated NADPH to the ground state, it emits BF at 460 ± 50 nm, which is distinguishable from their oxidized form NAD(P)^+^. NAD(P)H exists as an extended or folded state in an aqueous solution (Fig. 3) (Blacker and Duchen 2016). In the folded state, the fluorescent intensity decreases because of the quenching effect caused by the adenylyl moiety (Peng and Callender 2017). The fluorescence of NAD(P)H in the aqueous solution is in many cases amplified when it binds to the protein in an extended state (Fig. 4) (Hsu et al. 1967; Hwang et al. 2012; Lakowicz et al. 1992; Li and Lin 1996). However, BF is not always applicable to all NAD(P)-binding proteins. Indeed, NAD(P)H-dependent BF occurs by the simple binding of the cofactor to the protein, thereby indicating that the fluorescence is not related to an oxidative environment of cells. NAD(P)H-binding proteins can also be used for quantitating NAD(P)H.

### Enzymes can function as NAD(P)H-dependent BFPs

Some enzymes that exist in metabolic pathways can also function as BFPs upon binding with NAD(P)H. For example, the fluorescent intensity of bound NAD(P)H is four times higher when NAD(P)H binds to estradiol 17β-dehydrogenase at Ex/Em wavelengths of 340 nm and 436 nm, respectively (Li and Lin 1996). Malate dehydrogenase also amplifies the fluorescent intensity of bound NADH by 2–3 times, compared with free NADH in the aqueous solution without a shift in the emission wavelength (Lakowicz et al. 1992). The fluorescent intensity of NADH bound to lactate dehydrogenases is 2–3 times higher, compared with free NADH in solution, and its emission wavelength shifted from 465–470 nm to 445–450 nm (Cannon et al. 2021). The NADH fluorescence bound to alcohol dehydrogenase is two times higher than the free NADH at Ex/Em wavelengths of 337 nm and 445 nm, respectively (Konig et al. 1997). Conversely, the fluorescence of NADH bound to glyceraldehyde 3-phosphate dehydrogenase was decreased by ∼ 1.7 times, compared with its aqueous state (Velick 1958).

### Short-chain dehydrogenase/reductases

Short-chain dehydrogenase/reductase (SDR) is composed of 250 to 300 amino acid residues and includes a very large family of enzymes. Most members of this protein family are NAD(H)- or NADP(H)-dependent oxidoreductases (Gabrielli et al. 2022). SDRs commonly have a glycine-rich motif that is related to the cofactor binding to the protein (Graff et al. 2019). However, not all cofactor-binding proteins can emit detectable fluorescence, although some single-domain proteins have been reported as BFPs (Hwang et al. 2012; Su et al. 2001).

One SDR protein from *Vibrio vulnificus* CKM-1 (BFPvv) (Su et al. 2001) has been shown to bind to NADPH. BFPvv emits blue fluorescence with an emission peak of around 450 nm upon excitation by UV-VIS at 283 or 352 nm. It exhibits an enzymatic property, whereby it functions as an oxidoreductase against various aldehyde substrates (Polizzi et al. 2007), thereby making it unclear whether BFPvv and its derivatives can be used to quantify NADPH. Random mutational trials through directed evolution yielded one variant (BFPvvD7), whose BF intensity was increased by four times (Kao et al. 2011). Addition of mutations at three points to BFPvvD7 generated another derivative (BFPvvD8), whose fluorescence intensity was enhanced by 27%, compared with its parent protein. The emission spectra of these two derivatives have shifted to 440 nm. Some portion of the protein exists in a monomeric or dimeric state in solution in spite of its tetrameric structure in the crystalline state.

mBFP is another NADPH-dependent BFP that is isolated from the metagenomic DNA library and belongs to the SDR protein family (Hwang et al. 2012). mBFP exists as a homodimeric or homotetrameric protein in aqueous solutions, and its fluorogenic potential is moderately better than BFPvv. However, similar to BFPvv, it was rather obscure whether mBFP could be a reporter or quantitative tool with NADPH (You et al. 2019) owing to its capacity of oxidizing acetaldehyde and reducing nitrobenzaldehyde, which resulted in increased and decreased BF, respectively, in a time-dependent manner. Structure-guided mutation studies on the mBFP putative substrate binding site generated a variant (Seo et al. 2019); a single mutational change at the Tyr157 residue to histidine (Y157H) increased its fluorescence by 1.2 times, compared with wild-type mBFP. Interestingly, the fluorescent property of the Y157H variant was independent of the presence of putative substrates, such as acetaldehyde or nitrobenzaldehyde, and the NADPH levels remained constant during the measured time interval. This fluorescent property of the redox-free mBFP Y157H derivative was similarly maintained in the cytosolic-mimicking environment, which means that this variant can function as a potential in vitro and in vivo reporter for NADPH, regardless of intracellular metabolites.

Another recombinant SDR protein from *Burkholderia cenocepacia* J2315 is the closest structural homolog to mBFP and has also shown NADPH-dependent blue fluorescence, which is comparable to mBFP and BFPvv (Seo et al. 2019). In this background, other SDR proteins with similar structures and oligomeric states of mBFP have been suggested as being blue fluorogenic.

## Limitations of BFPs

### Limitations of GFP- and RFP-derived BFPs

Several GFP- or RFP-driven BFPs have been reported. However, their chromophores are formed in strictly aerobic conditions by intramolecular oxidative re-arrangement of three amino acids at a specific site following the biosynthesis of the entire polypeptide within the cell (Chia et al. 2019). Thus, their usage within aerobic systems is limited. Furthermore, the formation of chromophores in GFP derivatives is an event that requires a long maturation time to ensure their maximal fluorescence in vivo (Jalihal et al. 2019). Therefore, these limitations might make it problematic to perform real-time detection and monitoring of cellular events. Another potential issue is whether enhanced ROS formation and alterations in the expression of oxidative stress genes in bacterial and mammalian cells in response to GFP expression should be considered when interpreting any results obtained from experiments involving the expression of fluorescent proteins that probe redox-related mechanisms (Kalyanaraman and Zielonka 2017). These stresses could be closely linked with the oxidative cyclization of chromophores under aerobic conditions. Thus, determining the authenticity of this phenomenon will require more precise analysis.

Second, the relatively large size (typically ~ 27 kDa) of most FPs can compromise the function of co-expressed biomolecules or prevent labeled antibody reagents from accessing their targets. GFP-driven BFPs are often excited under near-UV wavelengths, which inevitably generate high auto-fluorescent background and poor signal-to-noise ratios (Tamura et al. 2021).

### Limitations of cofactor-dependent BFPs

In contrast to the oxygen-dependent formation of chromophores in GFPs and their derivatives, the fluorescence of NADPH-dependent BFPs is not related to the environmental redox state. Thus, this property can expand its biological application into anaerobic environments. However, the quantum yield of mBFP is rather low, ∼ 18% of that from GFPuv, and the photostability of mBFP is also relatively low and should be improved (Hwang et al. 2012). As shown in a case report that used mBFP, comparable imaging results were observed under specific conditions to those by GFP-derivative reporters; however, it is difficult to obtain reproducible results due to low photostability (Park et al. 2015). Thereafter, two-photon microscopy was employed to avoid photobleaching by mBFP–NADPH complexes or photodamage during long-term live cell imaging (Roshanzadeh et al. 2019).

Similar to GFP-like BFPs, the molecular weight (~ 25 kDa) of the cofactor-dependent BFPs is rather large, and they exist as oligomeric proteins in many cases, which can limit their application in various situations.

## Perspectives of BFPs

Applying more than two FPs, with different emission spectra, can provide advantages in the precise elucidation of cellular events. However, GFP along with its homologs and derivatives possess several defects: (1) the formation of chromophores through oxidative cyclization; (2) a BFP-exciting UV that also excites some cellular fluorogenic molecules; (3) relatively low intensity of BFPs, compared with GFP; and (4) relatively large size of the currently used FPs (more than 25 kDa). These suggest a necessity for the development of another BFP that functions regardless of the cellular redox state, which represents another mechanism with a weak background.

In these respects, NADPH-dependent BFPs are interesting since they function in aerobic and anaerobic conditions. NAD(P)H is a key molecule in cellular metabolism, redox reactions, biosynthesis, detoxification, and cellular defense mechanisms in living organisms (Selles Vidal et al. 2018; Xiao et al. 2018), which suggests that it can be applied to measure various cellular events. Notably, NAD(P)H could also serve as a biomarker for the development of drugs and diagnostic tools and for the industrial production of metabolites and chemicals (Croce et al. 2018; Kolenc and Quinn 2019). However, the innately low fluorescence intensity of the cofactor NADPH represents a bottleneck for the direct detection in vivo, although an advanced technique, such as FILM, was proven to be a plausible tool (Evers et al. 2018; Sharick et al. 2018). The application of NADPH-dependent BFPs in fluorometric assays can circumvent this problem since fluorescence spectroscopy can provide higher sensitivity and selectivity, which makes it possible to monitor NAD(P)H levels in real time (Goldbeck et al. 2018). It should be agreed that the fluorescent yield of NAD(P)H changes depending on the peripheral environment (Kolenc and Quinn 2019; Sun et al. 2021) since NAD(P)^+^ does not emit fluorescence. Therefore, despite monitoring of dynamic regulation using a genetically encoded sensor (Tao et al. 2017), the in vivo fluorescence-based techniques hardly assess the NAD(P)^+^/NAD(P)(H) redox ratio or the absolute amount of NAD(P)(H). Although these challenging works remain to be further improved, the first attempt for real-time monitoring of NADPH using mBFP in vivo was recently established in vivo by two-photon fluorescence microscopy (TPM) using animal cells as a model (Roshanzadeh et al. 2019). Obviously, genetically encoded mBFP sensors allow for long-term monitoring of intracellular NADPH flux in real time using TPM. The issue of a low fluorescence of NADPH-dependent BFPs with a high background can be circumvented by the development of BFPs with long wavelengths and optically modulated BFPs to selectively shift their signals to a unique detection frequency that is devoid of background. Presently, without these efforts, excitation of mBFP in the visible wavelength (750 nm) using TPM can solve some problems, including low photostability and autofluorescence. Moreover, the problem of causing stress in the ultraviolet range of the excitation wavelength and the problem of generating radicals can be partially avoided.

Although low photostability is avoided under the specific excitation route using TPM, the innate tetrameric nature of mBFP with high avidity toward NADPH could result in rapid and sensitive quantification of the cognate cofactor. The development of this prototypical technology means that it is possible to track not only the overall NADPH flux within cells but also the quantitative changes over time, both around or inside physiologically important sub-organelles, such as mitochondria and chloroplasts. As is well known, the production and consumption of NADPH in lactate metabolism in the mitochondria are known to be important indicators for the physiology of various cells, including cancers (Ying et al. 2021). In addition, since NADPH provides a very important biosynthetic driving force for the fundamental metabolic flux in this photosynthetic mechanism, real-time observation of the co-enzymatic flow in these organelles is expected to provide very decisive information on cell physiology, metabolism, disease, and defense mechanisms. Unfortunately, the non-targeted mBFP cannot be used to measure NADPH concentrations in organelles, such as mitochondria or chloroplasts. Instead, protein engineering of mBFP via fusion with a localization tag (signal sequence) will enable us to target the NADPH biosensor in specific organelles in live animal or plant cells, thereby paving a novel route to accessing NADPH flux in a real-time manner. The main determinant for this process is the fusion capacity (ability) since oligomeric NADPH-dependent BFPs have a fundamental problem, compared with the GFP-derived monomeric FPs. The modification of residues at the oligomeric interface can produce monomeric variants of NADPH-dependent BFPs, although this variant can bind a single NADPH, which results in a decrease in fluorescence or the loss of avidity. NADPH-dependent BFPs can be fused with proteins of low molecular weight or oligopeptide tags with an amorphous structure such as signal sequences (Glymenaki et al. 2022; Kreissl et al. 2023). However, most physiologically important proteins are multimers or have multiple domains, meaning that this option is not really feasible. Therefore, the discovery of a synthetic compound or peptide-based aptamer that mimics the NADPH-binding motif with similar surrounding residues to NADPH-dependent BFPs will provide an important turning point.

Alternatively, one approach for RNA imaging involves genetically encoding fluorescent RNAs through RNA mimickers of GFP (Ying et al. 2021). These mimicking RNA aptamers bind fluorophores resembling those found naturally in GFP and activate their fluorescence. These RNA–fluorophore complexes, including Spinach, Spinach2, and Broccoli, can be used to tag RNAs and image their localization in living cells (Sastre et al. 2023; Zhou and Zhang 2022). These molecules are sufficiently small and genetically encodable, meaning that they can function as a reporter to monitor in vivo events. Thus, RNA or DNA mimickers of mBFP that bind NADPH in vivo are expected to be technically feasible in the near future.
